# Supplementary material for: SARS-CoV-2-induced dysregulation in ADAR editing patterns persists post viral clearance in individuals with mild COVID-19
Source: Front Cell Infect Microbiol. 2026 Jun 2;16:1857062. doi: 10.3389/fcimb.2026.1857062 (PMC13269081; doi:10.3389/fcimb.2026.1857062)
Supplement: Supplementary file 1 [file SupplementaryFile1.docx]

**Supplementary Materials for: Aiswarya Mukundan Nair, Helen Piontkivska, “SARS-CoV-2 induced dysregulation in ADAR editing patterns persists post viral clearance in individuals with mild COVID-19”**

Supplementary materials and code are shared at <https://github.com/RNAdetective/Temporal_editing_in_SARSCoV2>, and at <https://zenodo.org/records/20174256>.

**List of supplementary tables and figures:**

Supp_Table_1. Supplemental_Table_1A: DESeq2 differential gene expression analysis results comparing Pre-infection samples to Mid-infection samples, filtered for log2Fold Change > |0.58| (Fold Change > 1.5) and an adjusted P value < 0.05.

Supplemental_Table_1B: DESeq2 differential gene expression analysis results comparing Pre-infection samples to Post-infection samples, filtered for log2Fold Change > |0.58| (Fold Change > 1.5) and an adjusted P value < 0.05.

Supplementary Table_1C: List of interferon stimulated genes differentially expressed mid-SARS-CoV-2 infection compared to pre-infection.

Supplemental_Table_1D: DESeq2 differential gene expression analysis results comparing Pre-infection samples to Mid-infection samples, filtered for log2Fold Change > |1| (Fold Change > 2) and an adjusted P value < 0.05.

Supplemental_Table_1E: DESeq2 differential gene expression analysis results comparing Pre-infection samples to Post-infection samples, filtered for log2Fold Change > |1| (Fold Change > 2) and an adjusted P value < 0.05.

Supp_Table_2. Supplemental_Table_2A: Reactome pathway over representation (OR) analysis results for genes differentially expressed mid-infection compared to pre-infection.

Supplemental_Table_2B: Reactome pathway over representation (OR) analysis results for genes differentially expressed post-infection compared to pre-infection.

Supplemental_Table_2C: Reactome pathway over representation (OR) analysis results for genes differentially expressed mid-infection compared to pre-infection (using stricter cut off Log2FC > |1| & adj p value <0.05.

Supp_Table_3. Supplemental_Table_3A: ADAR1 expression in TPM for each patient across pre-,mid-, and post-infection.

Supplemental_Table_3B: ADAR2/ADARb1 expression in TPM for each patient across pre-,mid-, and post-infection.

Supplemental_Table_3C: ADAR3/ADARb2 expression in TPM for each patient across pre-,mid-, and post-infection.

Supp_Table_4. Supplemental_Table_4A: Transcript level ADAR expression as DESeq2 normalized counts. Isoform transcript Ids were retrieved from Ensembl.

Supplemental_Table_4B: Transcript level ADAR expression data from DESeq2.

Supp_Table_5. Supplemental_Table_5: Total number of putative ADAR editing sites for each patient across pre-, mid-, and post-infection stages of SARS-CoV-2 infection.

Supp_Table_6. Supplemental_Table_6A: List of sites consistently edited pre-, mid-, and post-infection.

Supplemental_Table_6B: List of sites uniquely edited pre-, mid-, and post-infection.

Supplemental_Table_6C: REDIportal annotated pre-infection unique sites.

Supplemental_Table_6D: REDIportal annotated mid-infection unique sites.

Supplemental_Table_6E: REDIportal annotated post-infection unique sites.

Supp_Table_7. Supplemental_Table_7: Reactome pathways over representation (OR) analysis of genes incorporating confirmed unique editing sites (A) pre-, (B) mid-, and (C) post-infection.

Supp_Table_8. Supplemental_Table _8: Patient-wise overall ADAR editing levels across pre-, mid-, and post-infection stages of SARS-CoV-2 infection.

Supp_Table_9. Supplemental_Table_9: List of 45 individuals selected from the initial cohort of CHARM dataset with samples available across pre-, mid-, and post-infection stages.

Supp_Table_10. Supplemental_Table_10: Metrics of analyzed RNA-seq data from Bioproject PRJNA815324; GSE198449 (Sauerwald et al.,2022).

Supplemental Figure 1: Alterations in total number of putative ADAR edits mid- and post- infection compared to pre-infection.

Supplemental Figure 2: Elbow plots showing the total number of potential groups based on overall ADAR editing values in individuals post-infection.


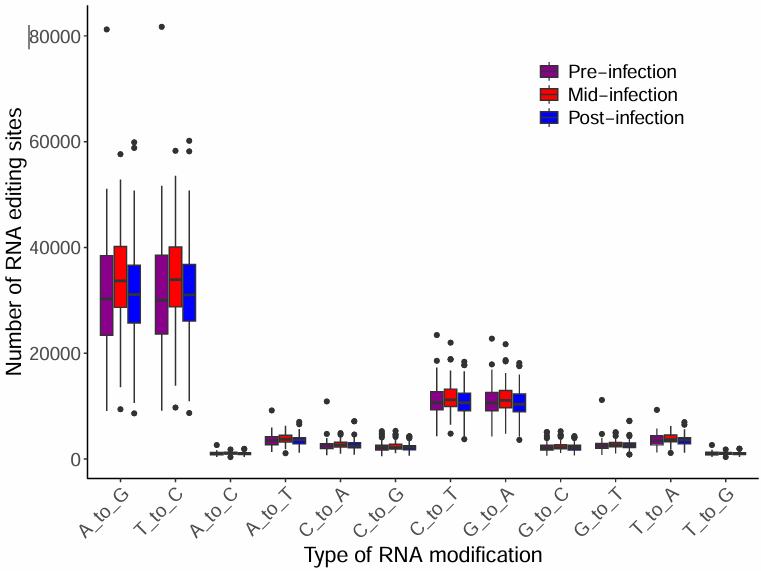


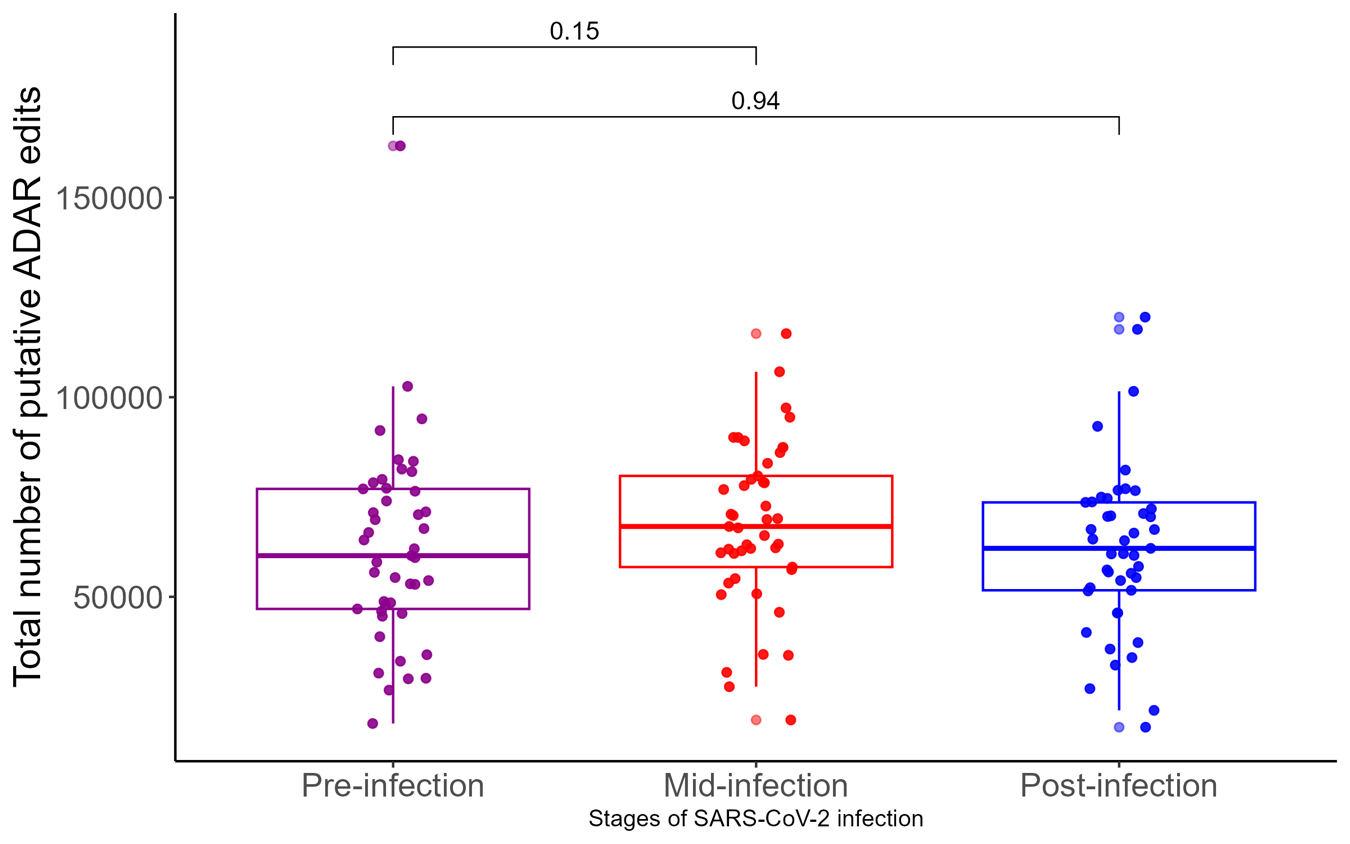


**Supplemental Figure 1**: **Alterations in total number of putative ADAR edits mid- and post- infection compared to pre-infection.** A-to-G and T-to-C substitution representing ADAR potential ADAR edits were the most abundant form of substitutions across all three stages of infection (A). While the total number of putative ADAR edits that includes both A-to-G and T-to-C substitutions increased mid-infection, the change did not achieve statistical significance (paired t-test, p value = 0.15). Similarly, there was no significant change in the number of putative ADAR edits post-infection compared to pre-infection (paired t-test, p value = 0.94) (B).


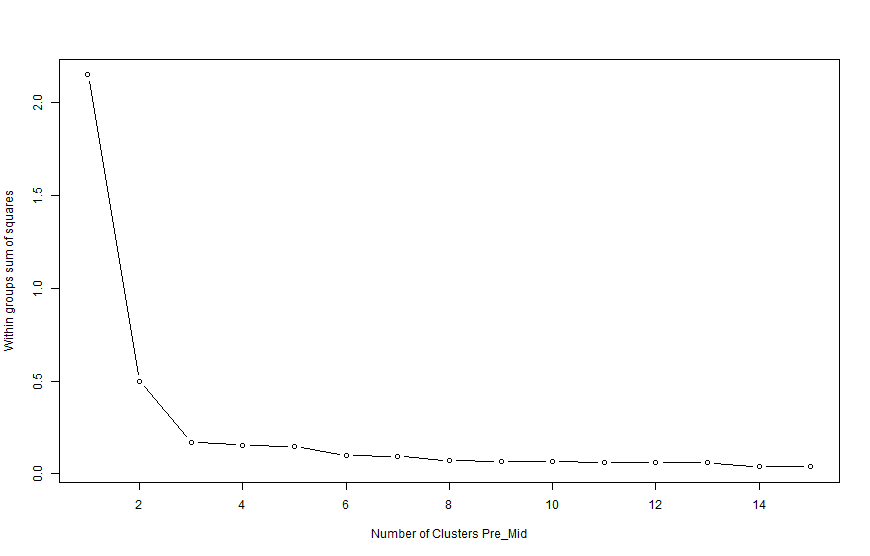

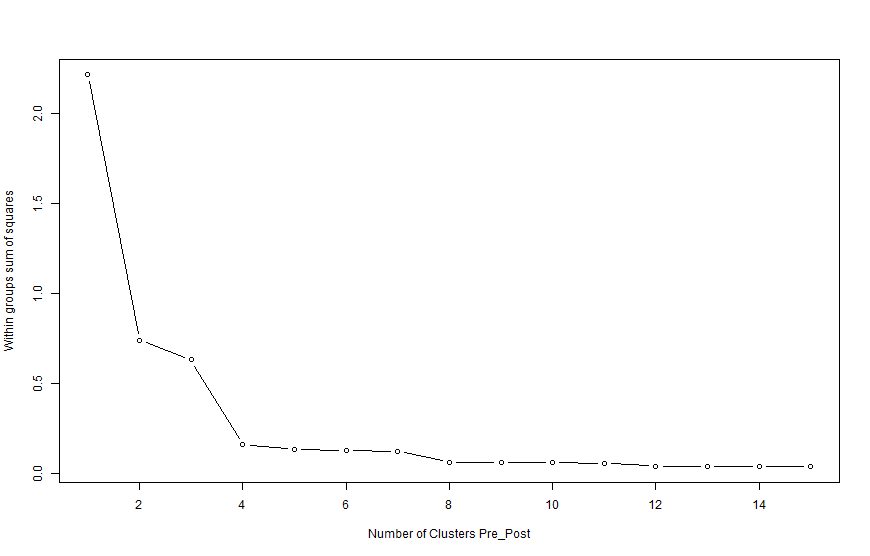


**Supplemental Figure 2**: Elbow plots showing the total number of potential groups based on overall ADAR editing values in individuals' pre- and mid-infection (2A) and pre- and post-infection (2B).
